# A novel approach to estimate the weight of food items based on features extracted from an image using boosting algorithms

**DOI:** 10.1038/s41598-023-47885-0

**Published:** 2023-11-29

**Authors:** Fotios S. Konstantakopoulos, Eleni I. Georga, Dimitrios I. Fotiadis

**Affiliations:** 1 Unit of Medical Technology and Intelligent Information Systems, Dept. of Materials Science and Engineering, University of Ioannina, Ioannina, Greece; 2 Foundation for Research and Technology-Hellas, Biomedical Research Institute, Ioannina, Greece

**Keywords:** Biomedical engineering, Nutrition, Lifestyle modification

## Abstract

Managing daily nutrition is a prominent concern among individuals in contemporary society. The advancement of dietary assessment systems and applications utilizing images has facilitated the effective management of individuals' nutritional information and dietary habits over time. The determination of food weight or volume is a vital part in these systems for assessing food quantities and nutritional information. This study presents a novel methodology for evaluating the weight of food by utilizing extracted features from images and training them through advanced boosting regression algorithms. Α unique dataset of 23,052 annotated food images of Mediterranean cuisine, including 226 different dishes with a reference object placed next to the dish, was used to train the proposed pipeline. Then, using extracted features from the annotated images, such as food area, reference object area, food id, food category, and food weight, we built a dataframe with 24,996 records. Finally, we trained the weight estimation model by applying cross validation, hyperparameter tuning, and boosting regression algorithms such as XGBoost, CatBoost, and LightGBM. Between the predicted and actual weight values for each food in the proposed dataset, the proposed model achieves a mean weight absolute error 3.93 g, a mean absolute percentage error 3.73% and a root mean square error 6.05 g for the 226 food items of the Mediterranean Greek Food database (MedGRFood), setting new perspectives in food image-based weight and nutrition estimate models and systems.

## Introduction

The management of an individual's daily dietary intake is a significant concern that impacts both low- and high-income nations. Inadequate dietary intake and unhealthy eating patterns have been identified as contributing factors to the development of malnutrition and a range of chronic diseases including obesity, diabetes, cancer, and cardiovascular diseases (CVDs)^1^. Malnutrition occurs when a person's daily intake of energy and nutrients is abnormally low or excessively high. Undernutrition (wasting, stunting, and underweight), which is defined by a lack of energy and nutrients, and overnutrition, which is characterized by an excess of energy and nutrients, are the two main types of malnutrition. Undernutrition is a contributing factor in 45% of the deaths of children under five in countries with low or middle income^2^. Malnutrition appears in one or more forms in every country, and it is one of the most significant challenges for both health and the economy worldwide. The National Center for Disease Control and Prevention estimates that 173 billion USD are spent on obesity-related medical care in the USA each year^3^.

Today, the widespread adoption of Artificial Intelligence (AI), the Internet of Things (IoT), and computer vision has enabled users to make use of food applications for monitoring and recording their dietary intake^4^. Recent studies have shown that AI-based applications are more popular among users compared to other recording methods^5^. Moreover, capturing food images through a smartphone provide the capability of continuous recording of health data in real time, as it offers the user a kind of interaction with the application, making it enjoyable to use. In contrast with traditional methods of calculating nutritional content, the widespread and increasing utilization of these applications has significantly accelerated their popularity. Nutritional assessment applications, such as Snap-n-eat^6^ and Carbs and Cals^7^, have been devised to automatically monitor, record, and compute an individual's daily dietary consumption without requiring their active participation. Two key components of these applications and systems include: (i) the dataset containing food images; and (ii) the subsystem responsible for estimating volume or weight of food.

The acquisition of food images and the development of a comprehensive database are associated with utmost importance in the context of nutrition assessment systems. This is due to their direct impact on the efficiency of artificial intelligence models and computer vision techniques employed in these systems. The way the images are taken directly depends on the methodology and techniques chosen to calculate the weight or quantity of the food. This process is known to be very demanding, often requiring a specific number of images, a specific methodology for capturing them, in many cases, a controlled environment, a calibrated camera, and often dedicated cameras designed specifically to capture food images. Actually, the process of determining the quantity or weight of a food image presents an enormous challenge, even for professionals in the field of nutrition. For instance, in the field of volume estimation, stereo vision techniques need the use of a minimum of two food images^8^, depth camera techniques require the usage of a specific device or sensor capable of accurately estimating the depth and distance of the captured image^9^, whereas deep learning techniques require a database of depth food images^10^.

Considering the challenges related to existing methods for calculating food quantities or weights from images, this study presents a novel dataset containing features extracted from annotated food images and a new approach for estimating food weight via the use of an advanced Machine Learning (ML) regression algorithm. In the present research, boosting regression algorithms were employed to determine the weight of a food image. The innovation of the proposed approach is the calculation of food weight through features extracted from a single image captured by a mobile phone camera. The weight was calculated based on various features extracted from annotated images, including the food weight, food area, reference object area, and other associated attributes. The annotations that have been extracted are utilized for the purpose of generating a dataframe that includes the features associated with the images. Next, a range of boosting regression models were developed and trained, achieving outstanding results that introduce novel insights into the field of food volume and weight estimation systems. Moreover, the recent study aims to address various challenges associated with image capture, image quantity, depth sensor utilization, and the constraints related to the types of food for which weight estimation is possible. Additionally, it can be integrated into dietary assessment systems and applications, thereby enhancing the precision of the food volume/weight estimation process. Finally, it is worth mentioning that the proposed model only needs one image in which the food it contains has previously been correctly segmented and classified to calculate its weight.

In summary, the main contributions of the current study include: (i) an annotated database of Mediterranean food images by weight, cuisine, food category and more, (ii) the creation of a dataset from features extracted from annotated food images, (iii) a unique optimization using the Optuna framework on well-known boosting algorithms trained on our generated dataset, (iv) an innovative approach that can calculate the weight of any food, regardless of its shape, texture, and form (liquid or solid), and (v) an approach that only needs just one image of the food and information extracted from its segmentation and recognition without additional terms and conditions.

## Related work

**Figure 1:**
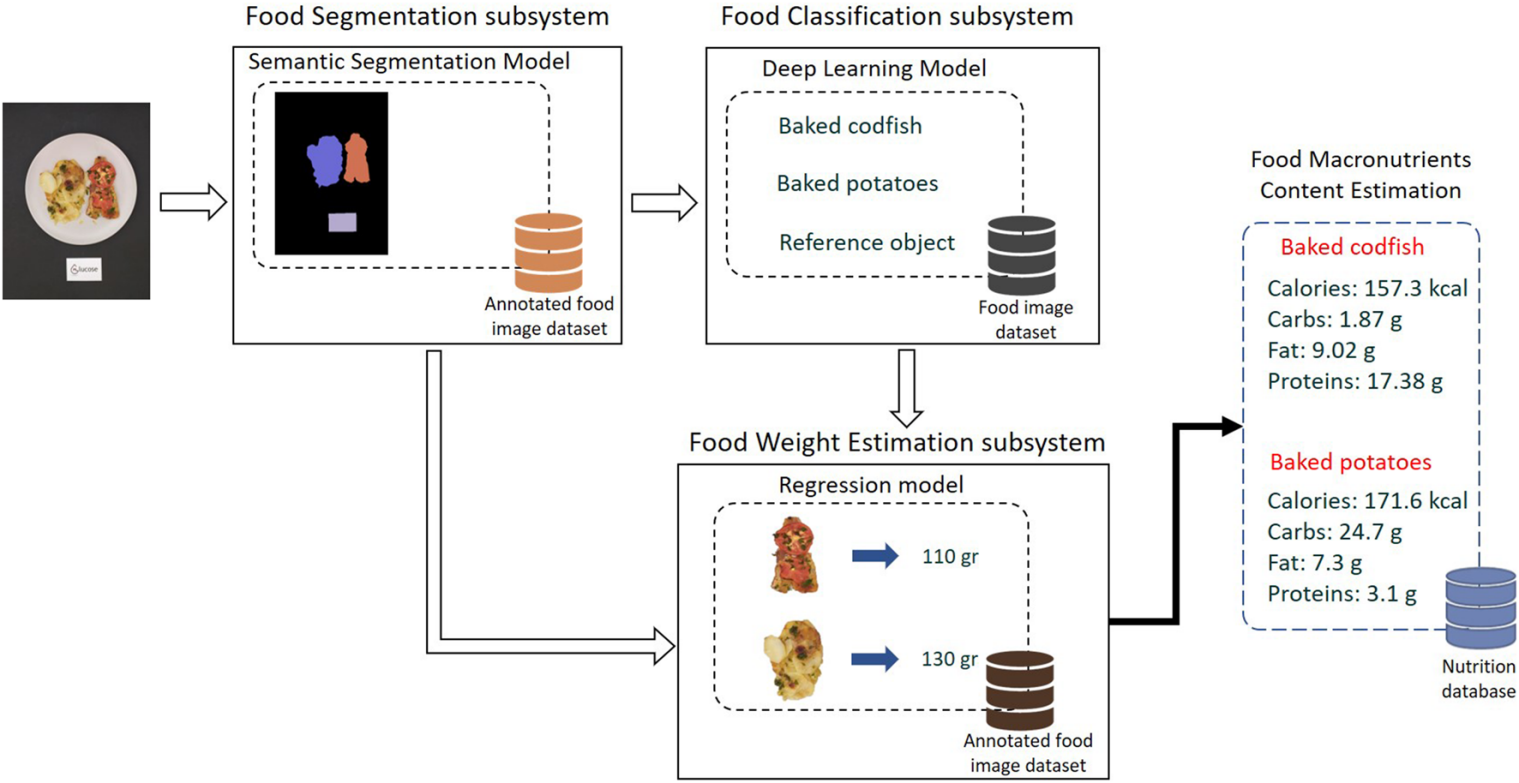
A dietary assessment system that includes the proposed methodology for estimating the weight of food.

The fundamental parts that constitute dietary applications and systems encompass two vital parts: (i) the food image dataset, which is an essential starting point for data analysis and model training, and (ii) the volume estimation subsystem, which provides a crucial part in accurately estimating the quantity or weight of food items^11^. Figure 1 shows a vision-based nutritional assessment system and its main components. Its main parts include those related to and developed for this research, including the food image database and the food weight calculation subsystem. The food image dataset plays a crucial role in the development of a reliable dietary assessment system and has a direct impact on the effectiveness of its subsystems. The characterization of a dataset can be determined based on two primary factors: the quantity of images and classes it encompasses, as well as the specific cuisine type it represents; the source from which the images are obtained; and the type of use for the image database (i.e., for segmentation, classification, or volume estimation tasks). The Food524DB^12^ dataset is comprised of a total of 247,636 food images that encompass a wide range of international cuisine. These images are categorized into 524 distinct food classes and were obtained from various existing databases. On the other hand, the UECFoodPix-Complete^13^ dataset specifically focuses on Japanese cuisine. It consists of 10,000 food images that have been annotated and categorized into 102 food classes. This dataset is particularly well-suited for tasks related to image segmentation. Depending on the method used to determine the meal's volume or weight, the image dataset for volume or weight estimate tasks may also include the depth map of the food images. Additionally, other details like food weight, camera features, or camera viewing angle are required to calculate the food volume^14^.

In vision-based dietary assessment systems, the most challenging tasks are volume or weight and nutrient estimation. The challenges associated with estimating the amount of food through image analysis for nutritional assessment systems are primarily attributed to the controlled environment required for food image capture, the need for multiple images, the difficulty in estimating the volume of the food with weak textural features, and the variability in dataset creation methods across different studies. These factors contribute to the complexity of accurately determining food quantities from images, as there are various interpretations and approaches employed by different systems in addressing this task. The categorization of current methods for estimating volume can be divided into five main categories^15^: (i) approaches based on stereo vision techniques^16^, (ii) approaches based on pre-build shape templates^17^, (iii) approaches based on perspective transformation^18^, (iv) approaches based on depth cameras^9^, and (v) approaches based on deep learning^19^. In general, each of the five approaches exhibits technical characteristics that impose certain limitations on their applicability. These limitations include the requirement for multiple images, a limited number of shape templates, dependence on wearable devices (e.g., depth camera), and the challenges associated with generating a three-dimensional food point cloud.

## Results

**Figure 2:**
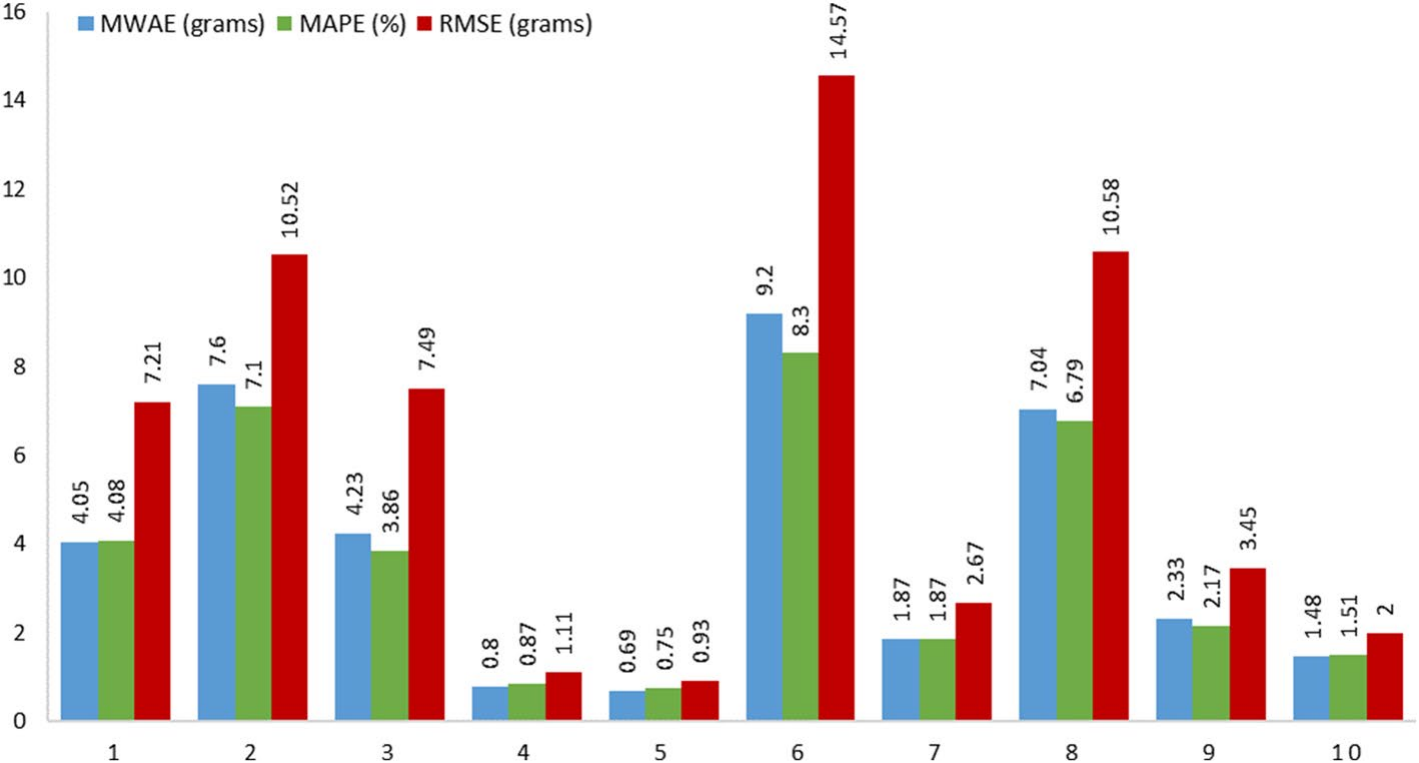
MWAE, MAPE, and RMSE metrics for each run of the XGBoost regression model.

**Figure 3:**
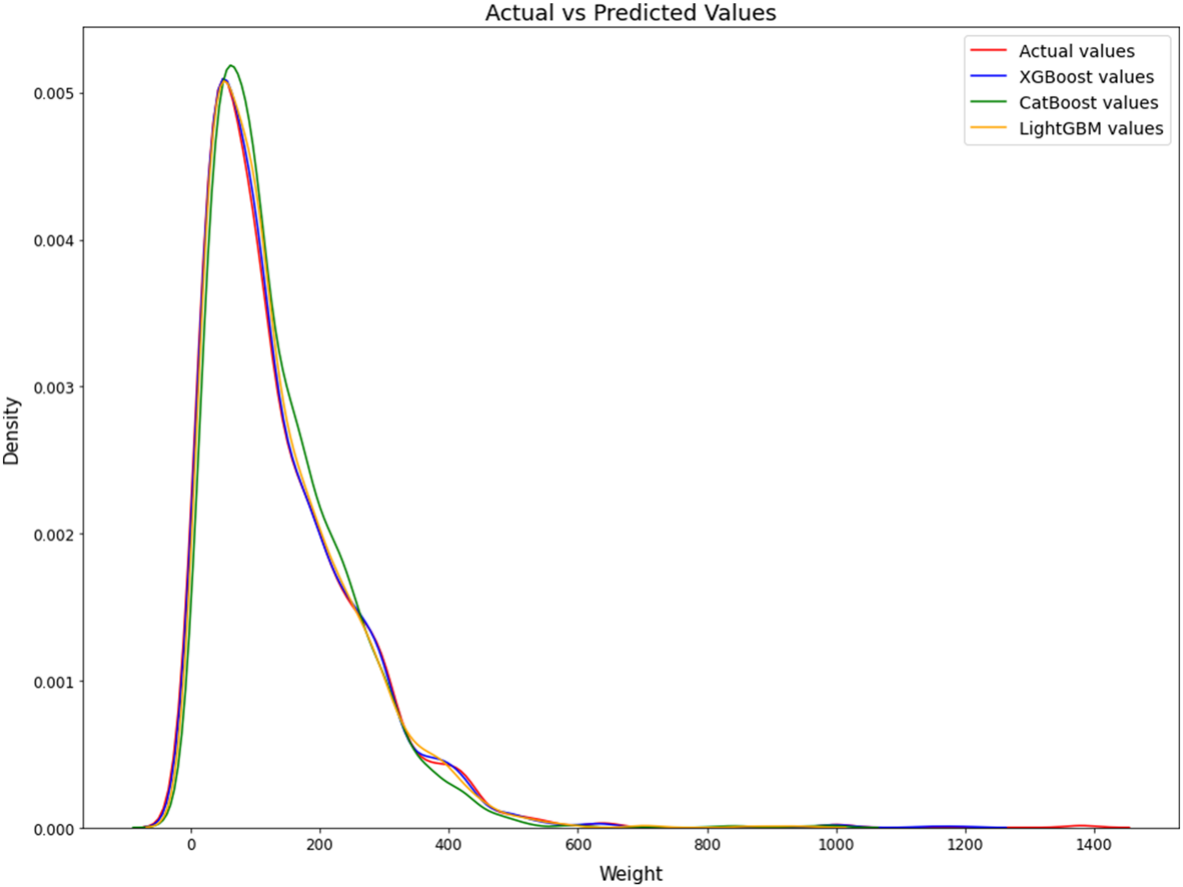
Density distribution of actual vs predicted values between the used boosting algorithms.

**Figure 4:**
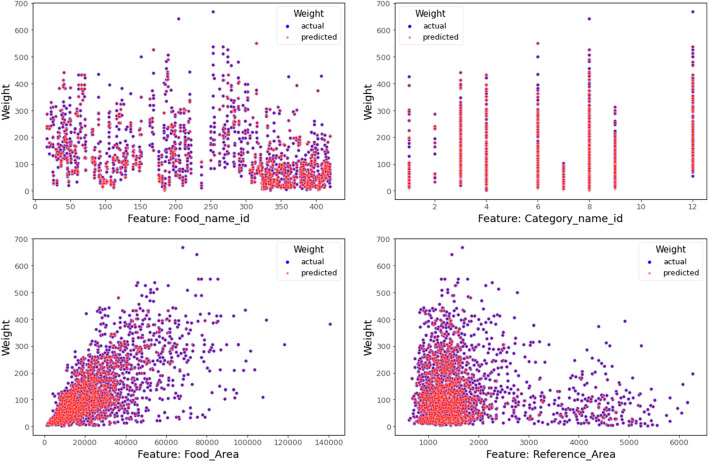
Predicted vs. actual values for food_name_id, category_name_id, food_area and reference_area features in the generated dataset using the XGBoost algorithm.

**Figure 5:**
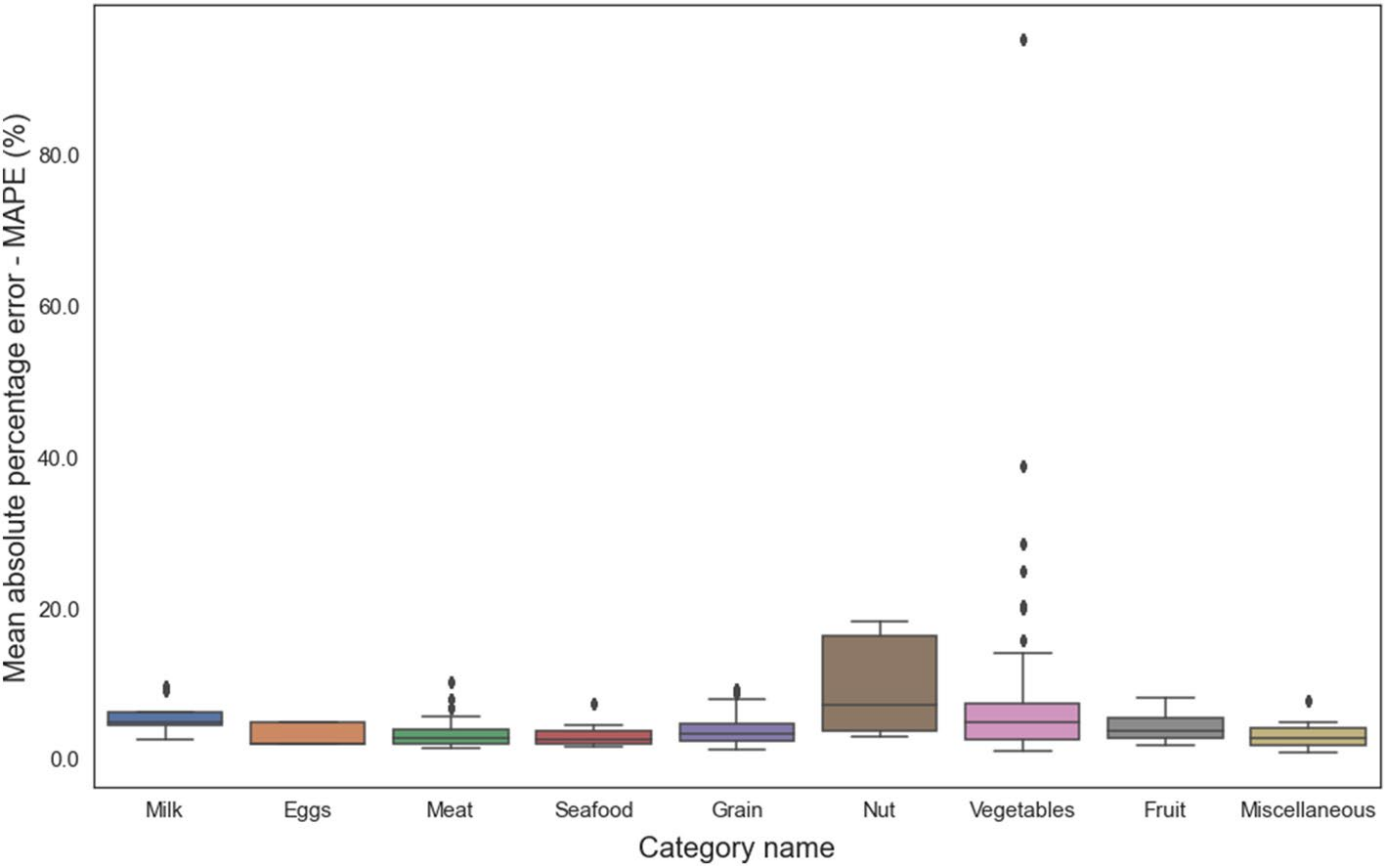
MAPE distribution across food categories.

The boosting regression models were validated using the validation subset. We performed the runs 10 different times for each model, employing a randomized approach to choose the training and validation sets. The average results and a comparison of tenfold cross validation are shown in Table 1 for the training models (XGBoost, CatBoost, and LightGBM) developed in this study. The proposed model, using the XGBoost algorithm, achieves a MWAE_overall_ of 3.93 g, a MAPE_overall_ of 3.73% and a RMSE_overall_ of 6.05 g per food item on the MedGRFood database. The model with the CatBoost algorithm achieves a MWAE_overall_ of 16.15 g, a MAPE_overall_ of 16.44% and a RMSE_overall_ of 22.19 g, and the final model with the LightGBM algorithm a MWAE_overall_ of 13.93 g, a MAPE_overall_ of 12.94% and a RMSE_overall_ of 21.26 g. The findings of this study are highly promising, since they present a novel approach to estimate the weight of food images that differs from the existing methods discussed in the relevant literature^15^ (Table 2). The outperforming results demonstrated by the model utilizing the XGBoost algorithm in comparison to the other two models are most likely due to XGBoost's ability to handle datasets with limited features as well as its improved ability to effectively optimize the hyperparameters. In Fig. 2, we show the MWAE, MAPE, and RMSE metrics for each run of the XGBoost regression model for the training and validation subset random splits. We notice that the best values in the evaluation indices are observed in the fifth time, while the worst values are observed in the sixth time. Figure 2 shows the superiority of the XGBoost algorithm in relation to the two other boosting algorithms employed. This is proven from the consistently superior performance of XGBoost, as its results above the average performance of the other algorithms. Furthermore, it confirms the very good overall outcomes achieved by the suggested pipeline on our generated dataset. Figure 3 presents the overall density distribution of the continuous actual and predicted values of the weight estimation models, where the superiority of the model based on the XGBoost algorithm is depicted (blue line). We observe that the distributions of actual and predicted values show more variation for foods weighing between 200 and 300 g and for foods weighing more than 700 g. In contrast, it can be observed that there is a convergence between the predicted and actual values, resulting in a lower variance, for food items that have a low weight. Similarly, this convergence is also observed for food items that weigh more than 300 g. In Fig. 4, we present the actual and predicted weight values compared to each of the dataset features generated for the proposed model. We observe that the largest residuals are for food items belonging to the category with id six, eight and twelve (grain, vegetable and miscellaneous products). In these categories, there are foods in liquid form, such as soups, where the exact calculation of their weight is a very difficult task due to the depth of the dish that contains them. In contrast, it is worth noting that foods that do not fall into the previously mentioned categories show improved accuracy in predicted weight measurement due to their more distinct shapes. Furthermore, looking at the predicted versus actual values relative to the area of the food in pixels, we observe a larger price deviation for foods with a larger surface area, which is also confirmed by the image associated with the feature reference to food area. In our analysis, it is clear that images featuring significant food or reference areas exhibit a greater degree of variability in weight calculation. The observed trend can be attributed to the utilization of a wide viewing angle during image capture. As a consequence, a larger number of pixels from the food or reference card area are included, thereby resulting in predicted values with greater variance. By implementing a protocol of slightly constraining the shooting angle and distance during the process of capturing images, it is possible to assume that the observed deviations in weight value prediction could be reduced, potentially leading to improved outcomes. Finally, Fig. 5 presents the distribution of MAPE across various food categories. We notice a large dispersion in vegetables, where there are foods with very little weight in which it is possible that there will be overlapping of their various pieces during photography (i.e., raw glistrida, spinach salad, parsley), so we are also led to a weight value prediction with a large deviation.

**Table 1:** Average results of the proposed boosting algorithms.

	MWAE_overall_ (g)	MAPE_overall_ (%)	RMSE_overall_ (g)
XGBoost (proposed model)	3.93	3.73	6.05
CatBoost	16.15	16.44	22.19
LightGBM	13.93	12.94	21.26

**Table 2:** Presentation of food volume and weight approaches including the proposed study.

Study	Approach	Techniques	Dataset	Results
Liu et al.^31^	Single view image with a reference object	Combining faster R-CNN, Grabcut, Median filtering, and CNN algorithm, they proposed a framework for estimating food volume	5 kinds of foods	Mean absolute error of each kind of food is less than 4.5%
Yang et al.^32^	Single-view RGB image	Estimates volume by computing the inner product of the modified MobileNetV2 probability vector and the reference volume vector	50 Chinese foods	11.6–20.1% mean relative volumetric error on 174–540 food images
Yuan et al.^33^	Multi-view method	3D reconstruction from multi-view RGB images	6 food items	0.83–5.23% measurement volume error
Lo et al.^19^	Deep learning view synthesis using a single depth image	Make use of 3D point cloud completion method to achieve accurate volume estimation	11 food items	15.3% mean volume estimation error
Proposed study	Single view image with a reference object	XGboost regression algorithm was employed to determine the weight of a food image, based on various features extracted from annotated food images	226 Mediterranean foods	Mean weight absolute error 4.05 g; mean absolute percentage error 4.08%; and root mean square error 7.21 g

## Discussion

In the field of food image databases, the application of deep learning techniques for the purpose of food recognition tasks has been observed to produce databases that aim to include a large number of images for each food category. The existing databases have several limitations in terms of the number of food classes they include, which is dependent upon the dietary preferences and practices of the researchers who are constructing the databases. The task of collecting food images and building food image databases has become less difficult nowadays, mainly due to the widespread practice of downloading and sharing images on social media platforms, which provides the ability to collect images from different sources. Nevertheless, the development of an extensive database that incorporates not only the nutritional information of food but also its constituent ingredients or weight remains a demanding task.

In this study, we presented an updated version of the MedGRFood database that includes more images and food categories with recorded food weight^11^. The MedGRFood food image database focuses primarily on Mediterranean cuisine, thus limiting its application to a wider range of culinary traditions. However, the process of annotating images and creating a dataset containing the unique features extracted from each annotated image provides an innovative perspective on how to approach similar problems. The dataset generated in this study represents an innovative effort in the field, as it is the first to present this structure. The proposed dataset was generated through the resulting question, "How can the problem of estimating the weight or amount of food be approached as a regression problem?". The previously mentioned question inspired the identification of the following features: food area, food reference area, food name id, category name id, and weight, which act as benchmarks for the proposed dataset. This dataset has significant potential to advance current methodologies used for image-based food weight estimation.

The task of estimating volume represents major difficulties in the context of vision-based dietary assessment systems. The use of depth cameras in the field of scale and quantity calculation, as well as in capturing multiple images for 3D reconstruction of food, presents certain constraints that limit their extensive adoption. Moreover, it is crucial to note that the application of food estimation methodologies based in geometric patterns allows for the calculation of volume estimation only for a limited number of food items characterized by identifiable geometric formats. The application of deep learning methods in the domain of food volume estimation has attracted significant interest in recent years due to its promising results. However, it has been observed through relevant literature^14^ that these techniques do not exhibit superior performance compared to the methodologies currently in use.

Table 2 presents the comparison of recent food volume or weight approaches with the proposed study. It is obvious that the proposed study is superior in terms of the number of foods for which their weight can be calculated, in that it requires only one image without additional devices and without a specific acquisition method, and in that it can be applied to both solid and liquid foods without limitations about the shape of the plate or the type of reference object that it needs. This study's innovative method for calculating food weight based on an annotated image is the reason for this distinction. The dataset that has been generated enables us to establish a correlation between the calculation of food weight and a regression problem, further allowing us to treat it as a food weight estimation problem rather than a food quantity estimation problem. To address this, we proceeded by building and training boosting regression algorithms. The outcomes obtained from the implemented model, utilizing the XGBoost algorithm, exhibit a notable advancement over the existing methodologies^14^. The decision to exclusively consider algorithms from the boosting family was based on their potential efficiency in addressing regression problems and their ability to surpass the performance of traditional ML and deep learning algorithms. They exhibit the ability to deal with multiple categorical features, demonstrating superior outlier handling capabilities compared to other algorithms. Additionally, boosting algorithms show reduced bias, mitigating the risk of overfitting, and they enable the optimization of regression models across a wide range of parameters. Also, although in the respective research studies they usually present the results of one metric, in our research we presented the results of all the metrics used in food volume or weight estimation tasks through images. In addition, although similar studies make a clear distinction and estimate the quantity of solid foods^16^, the present study offers a holistic approach without any distinction. This novel approach offers a promising solution to address the basic challenge of accurately estimating food weights through images. The methodology proposed requires the inclusion of just one image, preferably captured from a view from above or with a low viewing angle, and demands the accurate segmentation and classification of the food items present on the plate. This process will provide useful information about the food itself, its category, and finally the area of pixels covered by the food and the reference object. The next steps of our research include evaluating the proposed system on an external food dataset, as boosting algorithms tend to underperform in a range of values different from the one, they were trained on. Also, building and training more complex models for food weight estimation utilizing Convolutional Neural Network (CNN) and Long Short-Term Memory (LSTM) models are among our priorities.

## Conclusions

In this study, the architecture and overall concept of a model for estimating the weight of food by extracting features from annotated images were presented. The appropriate dataframe was created and the model is based on an augmented regression algorithm. The proposed methodology and model provide an innovative approach and solution to the problem of calculating food weight from images. By combining it with the database of nutrients and macronutrients^20^ and integrating it into a dietary assessment system, it aims to support health professionals in identifying dietary risks and consumers in following a healthy and balanced diet. Both perspectives play a significant role in the prevention of malnutrition, as well as several other diseases and conditions related to nutrition.

## Methods

### Food image dataset

**Figure 6:**
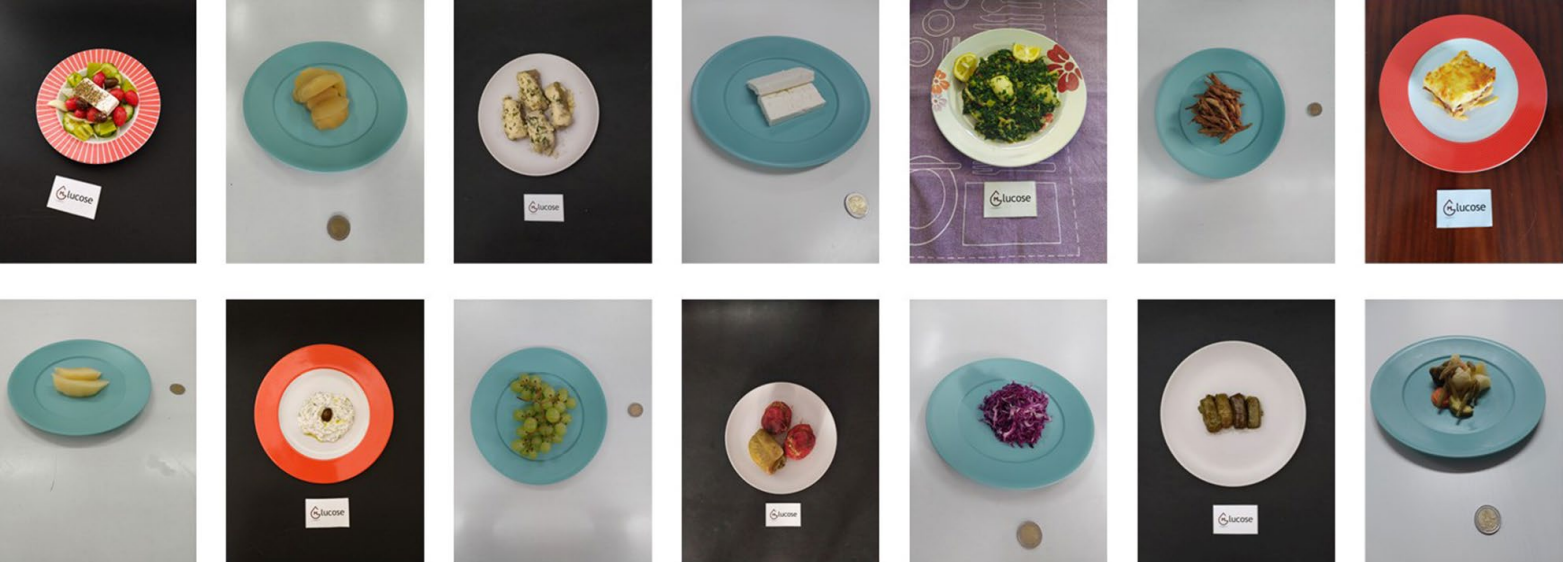
Examples of food images from the MedGRFood dataset used in this research.

**Figure 7:**
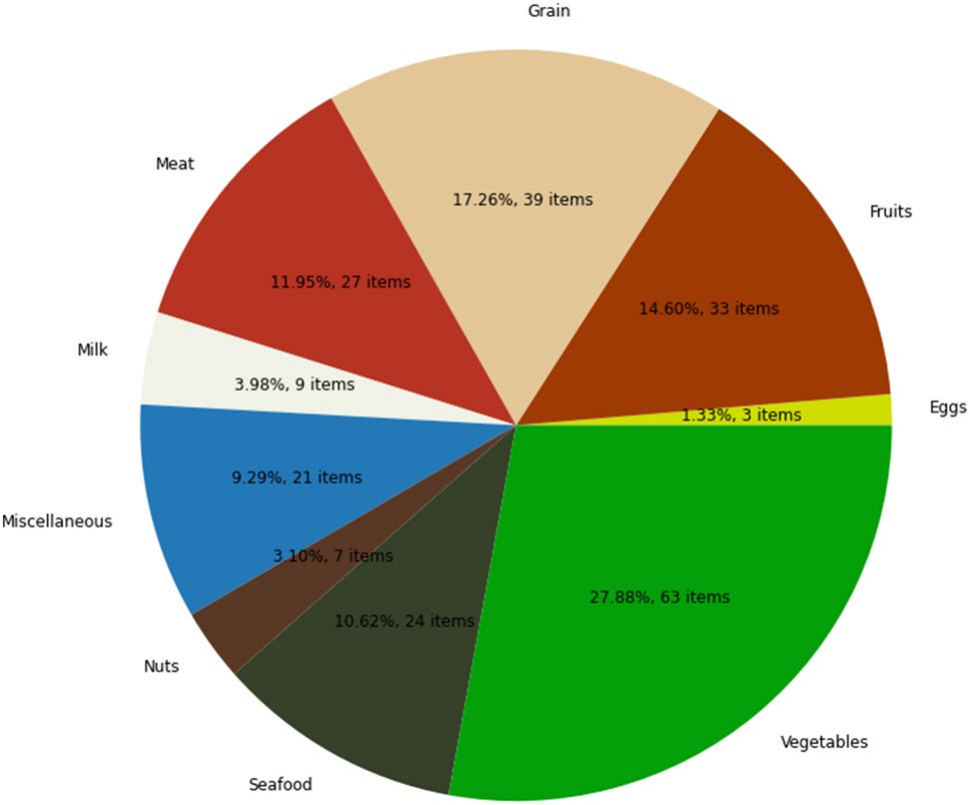
Distribution of food items for each food category in the MedGRFood database.

**Figure 8:**
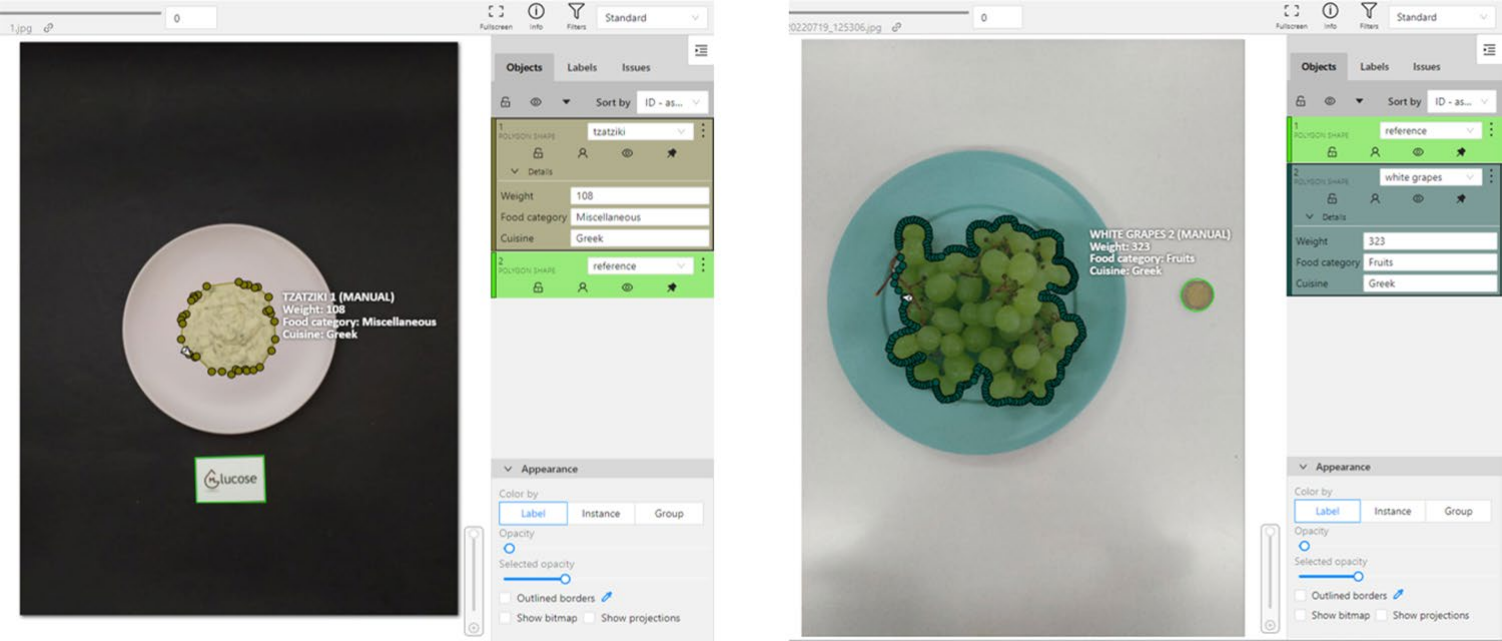
Annotated images using CVAT annotation tool with imported attributes.

The MedGRFood^21^ image database was utilized in the current study for both training and evaluating the weight estimation model. The MedGRFood dataset is a recently introduced database of food images specifically focused on Mediterranean cuisine. It comprises a total of 51,840 images, each representing a distinct Mediterranean dish, and these images are categorized into 160 classes, making it suitable for various classification tasks. Additionally, the dataset includes an additional subset of 23,052 food images, categorized into 226 categories with at least 100 images for each food. The entirety of the images have been systematically collected within a controlled environment, where a reference object (card or coin) has been placed next to the dish. This subset is particularly useful for tasks related to volume estimation and contains images of Mediterranean dishes, such as pastitsio, moussaka, seafood dishes, nuts, fruits, etc. (Fig. 6). Figure 7 shows the distribution of food items for each food category in the MedGRFood image database used in this study. In the context of this research work, it was necessary to systematically annotate the entire collection of 23,052 images within the selected subset. The annotations were accurately executed, focusing on several key aspects, including the categorization of the food, the specific name of the food item, the cuisine associated with the dish, the presence of any reference objects, and lastly, the weight of the food item in grams. The database includes a wide range of images for each food item, capturing different viewing angles and distances between the camera and the dish. The CVAT^22^ annotation tool, an open-source tool, was utilized for the purpose of food image annotation. Figure 8 shows examples of CVAT-annotated images and the attributes imported. Then, the annotated images were exported in COCO format^23^, generating a JSON file that, with appropriate processing, creates a two-dimensional data structure of dimensions 24,996 × 12 with the features of the images. The creation of the dataframe includes food items that consist of multiple pieces, resulting in a larger number of records compared to the total number of images available in the MedGRFood database. This dataframe comprises 24,996 records arranged as rows, each containing 12 different features represented as columns. Some of these features included are: the food name, food name id, category name, category name id, weight, food area in pixels, and the reference area in pixels, creating a unique dataset that includes food features which is suitable for training machine learning regression models. There is a direct connection between the fields food name and food name id, and the fields category name and category name id based on the Greek Composition dataset by the Hellenic Health Foundation^24^. For example, the food name “pastitsio” has food name id 274 and category name id 12. Table 3 shows the creation of the data structure of the annotated images exported in COCO format.

**Table 3:** Structure of the generated dataframe dataset.

Image id	Food name	Food name id	Category name	Category name id	File name	Width	Height	Food area	Reference area	Weight	Cuisine
88	Boiled greens	222	Vegetables	8	2.jpg	600	800	25,370	1992	103	Greek
73	Dolmades	116	Grain	6	63.jpg	600	800	25,424	1919	204	Greek
35	Fava	217	Vegetables	8	68.jpg	600	800	30,123	2349	91	Greek
14	Ouellette	20	Egg	2	87.jpg	600	800	58,388	1476	235	Greek
93	Pork souvlaki	317	Meat	3	15.jpg	600	800	11,867	11,031	74	Greek
77	Tzatziki	293	Miscellaneous	12	74.jpg	600	800	37,590	1142	245	Greek
82	Feta	372	Milk	1	25.jpg	600	800	12,051	1478	99	Greek
8	Fried gopa	331	Seafood	4	39.jpg	600	800	18,641	5970	69	Greek
95	Hazelnut	323	Nuts	7	56.jpg	600	800	29,737	1238	87	Greek
49	Melon	393	Fruits	9	75.jpg	600	800	16,628	1445	120	Greek

### Data manipulation

**Figure 9:**
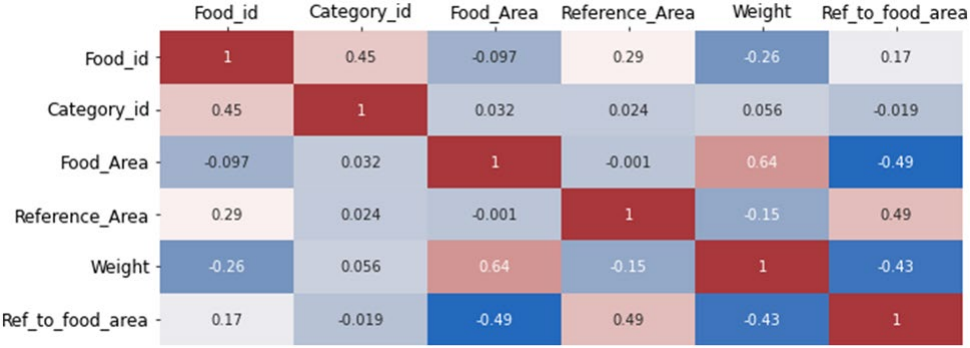
Correlation table between the features of the generated dataframe.

The next step in the proposed methodology includes data manipulation, where the data is methodically organized to enhance its readability, design, or structure. The process of data manipulation plays a crucial role in optimizing the utilization of information by systematically organizing raw data into a structured format. This procedure is required for improving productivity, identifying and analyzing patterns and trends, among other benefits. Since a large part of the images have been captured with a card as the reference object and the rest with a 2-euro coin as the reference object, the first step is to convert the reference area field so that all records refer to the 2-euro coin for reference. Knowing that the ratio between the areas covered by a reference card (8.5 cm × 5.5 cm) and the 2-euro coin is 8.9, we convert the records that have the reference object area of the card into a 2-euro coin. Next, we create a new field, the ratio of the reference area to the food area, which is unique for each type of food since it directly depends on the distance the image was taken and the perimeters of the areas of interest. Then, through the dataframe, a selection was made to exclude certain attributes, namely image id, food name, category name, file name, height, width, and cuisine. Consequently, the resulting dataframe contains a total of 24,996 records and 6 columns. Figure 9 illustrates the features and their respective associations within the generated dataframe.

### Boosting algorithms

Once the dataframe has been appropriately manipulated, a dataset that is suitable for machine learning regression techniques has been generated. The task of estimating weight can be defined as a regression problem, in particular for value forecasting. In this case, the requested value, indicated as y, represents the weight of the food and is considered the dependent variable, while the remaining features represent the independent variables. In the current study, the use of boosting machine learning algorithms was employed to calculate food weight. These algorithms were chosen due to their strong capabilities to solve regression problems, their ability to improve predictive accuracy, their capacity to handle diverse data types, and their flexibility in optimizing a range of loss functions. Boosting is a widely used ensemble learning technique in which a series of weaker learners are sequentially fit to a given dataset. This iterative process aims to improve the overall predictive performance of the ensemble by focusing on the instances that were previously misclassified. By iteratively adjusting the weights assigned to each instance, boosting effectively emphasizes the difficult-to-classify instances, allowing subsequent learners to focus on these challenging cases. This iterative nature of boosting enables the ensemble to learn from the mistakes made by previous learners, leading to a more accurate and robust final prediction. Each subsequent weak learner that is trained is designed with the objective minimize the errors that arise from the previous learner^25^.

#### Extreme gradient boosting

**Figure 10:**
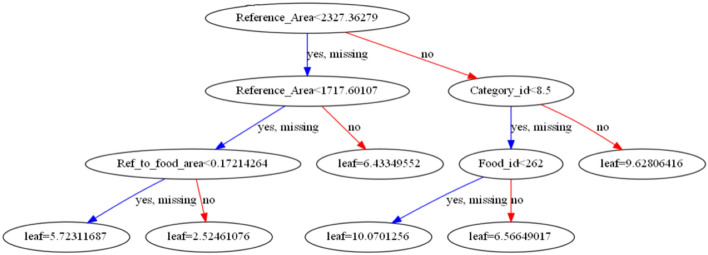
A decision tree structure of the proposed XGBoost algorithm.

The algorithm initially utilized for the computation of food weight was the Extreme Gradient Boosting^26^ (XGBoost) algorithm. XGBoost is an optimized implementation of gradient boosting (Table 4) that has received significant popularity and appreciation due to its efficiency and scalability. Also, XGBoost is a popular machine learning algorithm that presents a range of enhancements compared to the traditional gradient boosting technique. These advancements include the incorporation of regularization techniques, the ability to handle sparse data efficiently, the utilization of parallel computing for improved performance, and an outstanding accuracy that surpasses other machine learning algorithms in various predictive modeling cases. XGBoost works through an ensemble learning technique that utilizes the combination of multiple weak learners to construct a robust and powerful learner, and a training process that involves the construction of multiple decision trees. Finally, each tree is trained on a subset of the data, and the predictions from each tree are combined to form the final prediction. The structure of the proposed XGBoost regression algorithm is shown as a decision tree in Fig. 10. The numbers in the ellipses represent the feature thresholds defined during the decision tree’s construction.

**Table 4:**
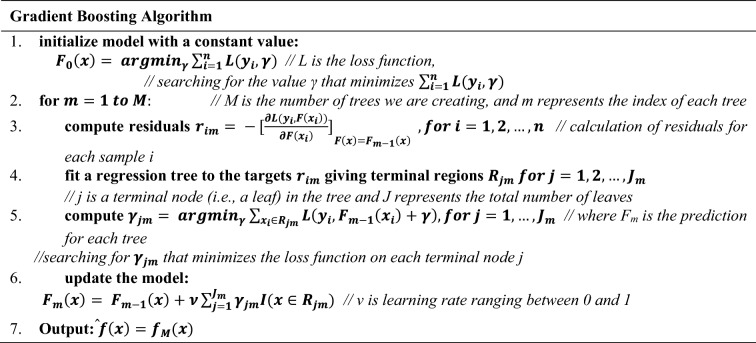
Pseudo code of gradient boosting algorithm.

#### Categorical boosting

The next algorithm employed in our study was the Categorical Boosting^27^ (CatBoost) algorithm. The CatBoost algorithm is a depth-wise gradient boosting technique that has been developed to address the challenges associated with effectively handling categorical features. The approach employed in this algorithm involves the utilization of a hybrid technique, specifically gradient boosting, in conjunction with one-hot coding. The utilization of this particular combination allows the efficient handling of categorical variables, consequently reducing the necessity for extensive preprocessing steps. The CatBoost algorithm incorporates a range of techniques to effectively address the issue of overfitting that can happen during the boosting process. The proposed approach incorporates regularization techniques, specifically "l2" and "l1" regularization, to prevent overfitting and enhance generalization. These regularization techniques are applied to the leaf values of the trees. Additionally, the method employs feature selection techniques (i.e., border count), which further contribute to the prevention of overfitting and generalization.

#### Light gradient boosting machine

The final boosting algorithm we used was the Light Gradient Boosting Machine^28^ (LightGBM). LightGBM is a distributed and efficient gradient-boosting framework (Table 4) that uses tree-based learning, that is designed to be highly efficient and scalable. The decision trees in LightGBM are constructed using a unique technique called "Leaf-wise" tree growth. In contrast to conventional depth-first approaches such as Depth-wise or Level-wise, where trees are expanded by dividing nodes at each level, LightGBM adopts a top-down approach by selecting the leaf nodes. The algorithm employs the strategy of selecting the leaf node with the highest delta loss for splitting. This approach leads to the generation of trees that are both more informative and deeper in structure. It is very fast in handling a large amount of data thus it is named as “light”. It introduces several innovative techniques, such as Gradient-based One-Side Sampling (GOSS) and Exclusive Feature Bundling (EFB), which enable it to handle large-scale data and high-dimensional feature spaces. Finally, LightGBM effectively addresses the issue of overfitting by integrating regularization techniques, specifically L1 and L2 regularization.

### Hyperparameters tuning

**Figure 11:**
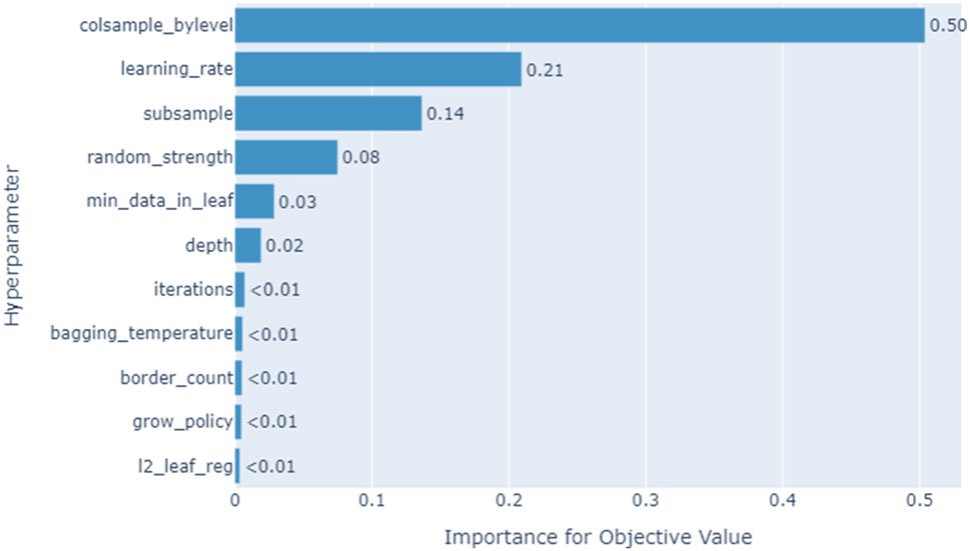
Hyperparameter importance of the CatBoost algorithm.

Hyperparameters refer to the adjustable variables that are used to determine the learning process of a machine learning model. The users demonstrate the ultimate authority to determine how the model acquires knowledge about a particular association between input data and related predictions. The optimization of a model's hyperparameters is a crucial step in addressing a particular problem, as it enables the development of an optimal model by identifying the most appropriate combinations of hyperparameters. The proposed model should have the ability to yield the best results by minimizing the loss function. To tune the hyperparameters of the proposed weight estimation models, we used the Optuna framework^29^. Optuna offers a convenient and efficient way to incorporate various advanced optimization techniques for the purpose of rapidly and effectively optimizing hyperparameters. By default, Optuna employs a Bayesian^30^ optimization algorithm (TPE). However, it offers the flexibility to seamlessly switch to alternative algorithms available within the Optuna framework. Figure 11 presents the hyperparameter importance of the proposed CatBoost algorithm.

### Training and evaluation

**Figure 12:**
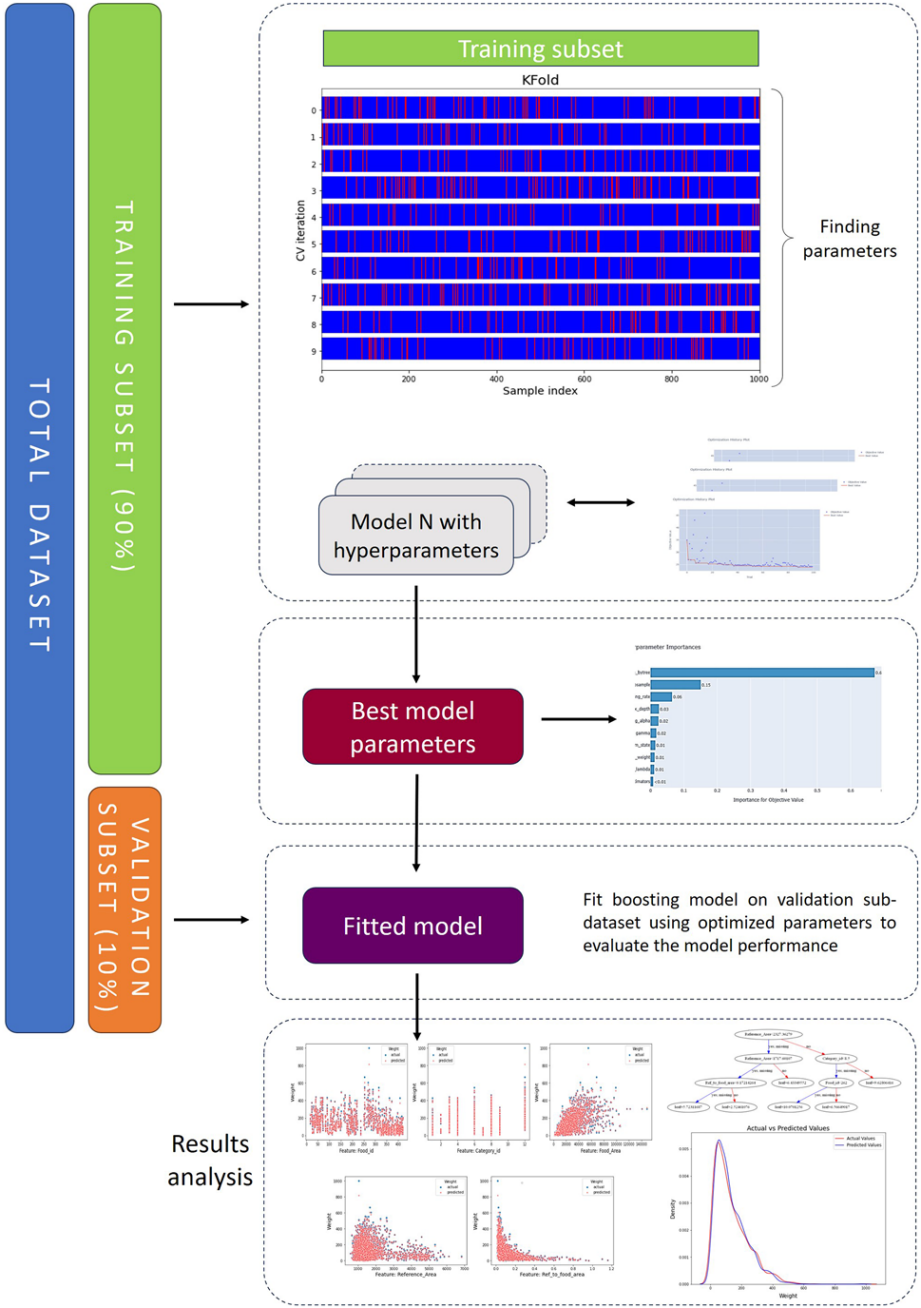
The proposed weight calculation pipeline during the training and validation steps.

**Figure 13:**
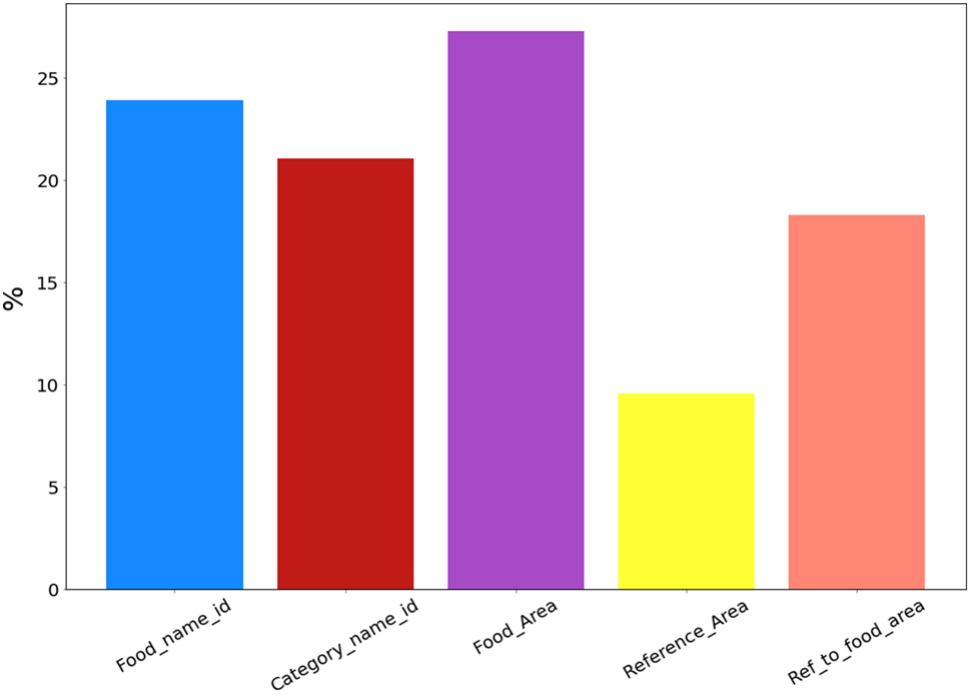
Feature importance of the XGBoost algorithm.

To train and evaluate the boosting algorithms, we split the data into training and validation subsets. For the validation subset, we randomly selected 10% of the records from each food after first shuffling the existing records for each of them. Thus, two sub-datasets are obtained from the existing dataset, the first containing 22,527 records used for training and evaluation and the second with 2469 records used for validation. Model testing was performed on validation subset that were not considered during the training phase and, consequently, did not influence the feature selection process. To account for randomness, obtain reliable and stable results, and ensure the robustness of our model evaluation, we performed the runs 10 different times with a random selection of the training and validation sets each time. Next, we performed a tenfold shuffle cross-validation (cv) on the training sub-dataset. Figure 12 shows the overall pipeline of the proposed weight calculation methodology during the training and validation steps. The primary objective of cross-validation is to assess the generalization performance of a machine learning model by evaluating its predictive capabilities on unknown data. Moreover, this technique is employed to identify potential issues such as overfitting or selection bias, as well as to provide valuable insights into the generalization capabilities of the model when applied to an independent dataset. In addition, early stopping techniques are employed to reduce the issue of overfitting in the training data. This is achieved by continuously evaluating the performance of the boosting models during the training process using a test dataset. If the performance on the test dataset doesn't show any improvement after a certain number of training iterations, the training procedure is stopped. For the training sub-dataset in each cv fold, we created additional features separately for the train and test subsets in order to improve the performance of the boosting algorithms. These additional features are for each food item: the average weight, the average food area, the average reference area, the weight standard deviation, and the ratio of the average reference area to the average food area. Figure 13 shows the feature importance for the proposed XGBoost model. We observe that all features affect the training of the XGBoost algorithm almost the same, except the Reference_area feature, which clearly affects less.

As evaluation metrics for the proposed model, we used for each food item the Mean Weight Absolute Error—MWAE:

1 $$MWAE= \frac{1}{n}\sum_{i=1}^{n}\left|{W}_{pred}- {W}_{real}\right|,$$

the Mean Absolute Percentage Error—MAPE:

2 $$MAPE= \frac{1}{n}\sum_{i=1}^{n}\left|\frac{{W}_{pred}- {W}_{real}}{{W}_{real}}\right|\times 100,$$

and the Root Mean Square Error—RMSE:

3 $$RMSE= \frac{1}{n}\sqrt{\sum_{i=1}^{n}{({W}_{pred}-{W}_{real})}^{2}},$$

where W_pred_ is the predicted weight of food, W_real_ is the real weight, and n represents the corresponding records for each food item present in the generated dataset. In total, we estimate the weight of 226 different dishes from the MedGRFood image dataset, using the evaluation metrics:

4 $${MWAE}_{overall}= \frac{1}{226}\sum_{i=1}^{226}{MWAE}_{i},$$

5 $${MAPE}_{overall}= \frac{1}{226}\sum_{i=1}^{226}{MAPE}_{i},$$

and,

6 $${RMSE}_{overall}= \frac{1}{226}\sum_{i=1}^{226}{RMSE}_{i}.$$

### Implementation

The workflows were executed under the high-performance computing infrastructure (HCI) which has been explicitly designed for data intensive tasks as part of the PRECIOUS project. The HCI currently includes 576 Intel(R) Xeon(R) Gold 5220R physical cores, 86000 CUDA cores, 4.6 TB RAM, and 0.5 PB raw storage. Also, we used the Python programming language to implement the dietary assessment system in the Anaconda environment, installing appropriate libraries for the implementation of the food weight estimation system.

## Data Availability

The data that support the findings of this study are available from the MedDietAgent and GlucoseML consortiums, but restrictions apply to the availability of these data, which were used under license for the current study and are not publicly available. Data are, however, available from the authors upon reasonable request and with the permission of the MedDietAgent and GlucoseML consortiums.

## References

[1] Himmelgreen D (2022). Using syndemic theory to understand food insecurity and diet-related chronic diseases. Soc. Sci. Med..

[2] World Health Organization, *Malnutrition*, https://www.who.int/health-topics/malnutrition (2021), (Accessed 9 June 2021).

[3] National Center for Chronic Disease Preventation and Health Promotion (NCCDPH), *Poor Nutrition*, https://www.cdc.gov/chronicdisease/resources/publications/factsheets/nutrition.htm, (Accessed 8 Sept 2022).

[4] Konstantakopoulos, F. S. *et al.* In *2022 44th Annual International Conference of the IEEE Engineering in Medicine & Biology Society (EMBC).* 1432–1435 (IEEE). 10.1109/EMBC48229.2022.9871732 36085710

[5] Carter MC, Burley VJ, Nykjaer C, Cade JE (2013). Adherence to a smartphone application for weight loss compared to website and paper diary: Pilot randomized controlled trial. J. Med. Internet Res..

[6] Zhang W, Yu Q, Siddiquie B, Divakaran A, Sawhney H (2015). “snap-n-eat” food recognition and nutrition estimation on a smartphone. J. Diabetes Sci. Technol..

[7] Chen J (2017). The use of smartphone health apps and other mobile h ealth (mHealth) technologies in dietetic practice: A three country study. J. Hum. Nutr. Diet..

[8] Konstantakopoulos, F., Georga, E. I. & Fotiadis, D. I. In *2021 IEEE 21st International Conference on Bioinformatics and Bioengineering (BIBE).* 1–4 (IEEE).

[9] Ando, Y., Ege, T., Cho, J. & Yanai, K. In *Proceedings of the 5th International Workshop on Multimedia Assisted Dietary Management.* 76–81. 10.1145/3607828.3617789 PMC10823382 38288389

[10] Lo, F. P. W., Sun, Y., Qiu, J. & Lo, B. In *2019 IEEE 16th International Conference on Wearable and Implantable Body Sensor Networks (BSN).* 1–4.

[11] Konstantakopoulos FS, Georga EI, Fotiadis DI (2023). An automated image-based dietary assessment system for mediterranean foods. IEEE Open J. Eng. Med. Biol..

[12] Ciocca, G., Napoletano, P. & Schettini, R. In *International Conference on Image Analysis and Processing.* 426–434 (Springer).

[13] Okamoto, K. & Yanai, K. In *International Conference on Pattern Recognition.* 647–659 (Springer).

[14] Lo FPW, Sun Y, Qiu J, Lo B (2020). Image-based food classification and volume estimation for dietary assessment: A review. IEEE J. Biomed. Health Inform..

[15] Konstantakopoulos FS, Georga EI, Fotiadis DI (2023). A review of image-based food recognition and volume estimation artificial intelligence systems. IEEE Rev. Biomed. Eng..

[16] Dehais J, Anthimopoulos M, Shevchik S, Mougiakakou S (2017). Two-view 3D reconstruction for food volume estimation. IEEE Trans. Multimedia.

[17] Fang, S., Liu, C., Zhu, F., Delp, E. J. & Boushey, C. J. In *2015 IEEE International Symposium on Multimedia (ISM).* 385–390 (IEEE). 10.1109/ISM.2015.67 PMC5035274 27672682

[18] Yang Y, Jia W, Bucher T, Zhang H, Sun M (2019). Image-based food portion size estimation using a smartphone without a fiducial marker. Public Health Nutr..

[19] Lo FP-W, Sun Y, Qiu J, Lo BP (2019). Point2Volume: A vision-based dietary assessment approach using view synthesis. IEEE Trans. Ind. Inform..

[20] Gebhardt, S. *et al.* USDA national nutrient database for standard reference, release 21. (J United States Department of Agriculture, Agricultural Research Service, 2006).

[21] Konstantakopoulos, F. S., Georga, E. I. & Fotiadis, D. I. In *2021 43rd Annual International Conference of the IEEE Engineering in Medicine & Biology Society (EMBC).* 1740–1743 (IEEE). 10.1109/EMBC46164.2021.9630481 34891623

[22] Computer Vision Annotation Tool (CVAT) v. 2.2.0 (2022).

[23] Lin, T.-Y. *et al.* In *European Conference on Computer Vision.* 740–755 (Springer).

[24] Trichopoulou A, Soukara S, Vasilopoulou E (2007). Traditional foods: A science and society perspective. Trends Food Sci. Technol..

[25] Bentéjac C, Csörgő A, Martínez-Muñoz G (2021). A comparative analysis of gradient boosting algorithms. Artif. Intell. Rev..

[26] Chen, T. *et al.* Xgboost: extreme gradient boosting. *R package version 0.4-2* vol. 1, 1–4 (2015).

[27] Hancock JT, Khoshgoftaar TM (2020). CatBoost for big data: an interdisciplinary review. J. Big Data.

[28] Ke, G. *et al.* Lightgbm: A highly efficient gradient boosting decision tree. *Adv. Neural Inf. Process. Syst.* **30** (2017).

[29] Akiba, T., Sano, S., Yanase, T., Ohta, T. & Koyama, M. In *Proceedings of the 25th ACM SIGKDD International Conference on Knowledge Discovery & Data Mining* 2623–2631 (Association for Computing Machinery, 2019).

[30] Wu J (2019). Hyperparameter optimization for machine learning models based on Bayesian optimization. J. Electron. Sci. Technol..

[31] Liu, Y. *et al.* In *Proceedings of the 2020 the 4th International Conference on Innovation in Artificial Intelligence* 84–89 (Association for Computing Machinery, 2020).

[32] Yang Z (2021). Human-mimetic estimation of food volume from a single-view RGB image using an AI system. Electronics.

[33] Yuan D (2021). An automatic electronic instrument for accurate measurements of food volume and density. Public Health Nutr..

